# Lifting the Standards: A Systematic Review of Aesthetic Scarless Breast Surgery

**DOI:** 10.7759/cureus.109131

**Published:** 2026-05-18

**Authors:** Emily R Stack, Jared Garfinkle, Erin N Burns, Colby V Spongberg, David Steckler

**Affiliations:** 1 Surgery, William Carey University College of Osteopathic Medicine, Hattiesburg, USA; 2 Surgery, Rowan-Virtua School of Osteopathic Medicine, Stratford, USA; 3 Surgery, Touro College of Osteopathic Medicine, Great Falls, USA; 4 Plastic Surgery, Mississippi Center for Plastic Surgery, Ridgeland, USA

**Keywords:** breast aesthetic surgery, breast surgery, mastopexy techniques, minimally invasive surgery, scarless surgery

## Abstract

Scarless breast surgery techniques, such as radiofrequency-assisted mastopexy, transaxillary and transumbilical augmentation, and liposuction-based reductions, have gained popularity for delivering aesthetic results while minimizing visible scarring. However, evidence on outcomes and complication rates across techniques remains variable. A systematic review of 11 studies with 1,517 patients undergoing scarless aesthetic breast procedures was conducted. Data on surgical techniques, patient satisfaction, anatomical changes, and complications were extracted. Outcomes were assessed through photographic reviews, global aesthetic scales, and objective measurements of nipple and areolar position. The average patient age was 34 years. Radiofrequency-assisted mastopexy reported a mean nipple lift of 1.2 cm, while the “Compass Rose” suture technique achieved an average areolar width reduction of 11 mm. Transaxillary and transumbilical augmentations demonstrated patient satisfaction rates of ≥90%. Reported complications were generally low and included hematoma (n=2), capsular contracture (n=2), implant malposition (n=1), and minor wound-related issues. Overall, complication rates were similar to or lower than those associated with traditional augmentation and mastopexy techniques. Scarless aesthetic breast procedures provide high patient satisfaction and measurable improvements in breast contour and nipple position, with complication rates comparable to conventional approaches. Future research should focus on standardized reporting and long-term outcomes to better define the efficacy and safety of these minimally invasive techniques.

## Introduction and background

“Scarless” breast surgery encompasses a wide range of aesthetic procedures that aim to hide scarring by creating incisions in concealed areas or to reduce scarring altogether through minimally invasive techniques. The most common of these techniques involve alternatives to breast augmentation, reduction, and mastopexy. The approaches and instrumentation used for these surgeries have evolved significantly over the past several decades to enhance cosmetic appeal [1,2].

The demand for scarless procedures has sharply increased as they have become more widely available. A 2024 article reports that aesthetic procedures have increased 40% in volume since 2021 [3]. The American Society of Plastic Surgeons’ annual report shows that over 600,000 cosmetic breast procedures were performed in the United States in 2024, and all major categories - augmentation, lift, reduction, gynecomastia revision, and implant removal - saw an increase from the year prior [4]. Patients prefer shorter recovery times, fewer complications, and improved aesthetic outcomes, all of which can be associated with minimally invasive approaches [5].

Three main approaches have been taken to accomplish minimal scarring in aesthetic breast surgery, which are axillary, inframammary, and periareolar [6-11]. Beyond the incision location, these scarless procedures vary substantially in instrumentation, graft type, and suture techniques or materials. Mastopexies may incorporate advanced technology such as radiofrequency energy to accomplish the lift [12,13]. Breast augmentations use several different techniques to reduce scarring such as autologous grafting, utilizing endoscopic tools, or using non-traditional approaches such as transumbilical entry [14-16]. Liposuction has now been applied in both breast reductions and gynecomastia revisions [17,18]. These are a few examples of the variants of common procedures that will be evaluated in this systematic review. 

Despite the interest and innovation in scarless aesthetic breast surgery, significant discrepancies in reporting remain, making outcome comparisons difficult. The BREAST-Q is a standardized survey that includes an assessment of the patient’s satisfaction with their breasts, overall outcome, process of care, psychosocial well-being, physical well-being, and sexual well-being [19]. It was created to avoid inconsistencies in reporting; however, many articles regarding scarless breast surgery have neglected to use the BREAST-Q. Aesthetic outcomes are often assessed through subjective methods, and key clinical outcomes such as operative time, BMI, complication rates, revision frequency, and recovery duration are inconsistently reported. These limitations prevent generalizability as well as evidence-based recommendations. Therefore, there is a need for a strategic review of scarless breast literature and outcomes in order to truly compare the scarless breast surgery outcomes to their traditional, scar-leaving counterparts. 

This systematic review and meta-analysis aims to evaluate outcomes of scarless aesthetic breast surgery with focus on augmentation, reduction, gynecomastia revision, and mastopexy. It will analyze surgical approaches and techniques in relation to patient satisfaction outcomes, complication rates, recovery time, and operative duration. By synthesizing the available data, this review seeks to gauge the success of scarless breast procedures, investigate the need for outcome reporting standardization, and identify gaps in the research.

## Review

Methods

Protocol and Registration 

This systematic review was conducted with the Preferred Reporting Items for Systematic Reviews and Meta-Analyses (PRISMA) guidelines. A study protocol outlining search strategy, eligibility criteria, and data extractions was developed prior to initiating the review process. The study protocol was registered on the international register of systematic reviews (PROSPERO) under the ID: CRD420251128138, on August 25, 2025, and is available online at (https://www.crd.york.ac.uk/PROSPERO/view/CRD420251128138).

Information Sources and Search Strategy

A comprehensive search of MEDLINE (Ovid, Ovid Therapeutics, New York, NY, USA), Embase (Elsevier, Amsterdam, The Netherlands), the Cochrane Central Register of Controlled Trials (Wiley, Hoboken, NJ, USA), and ClinicalTrials.gov (National Library of Medicine, Bethesda, MD, USA), was performed, using a controlled vocabulary related to scarless breast surgery, minimally invasive mastopexy, radiofrequency-assisted breast lift, transaxillary augmentation, and liposuction-based breast reduction. 

Eligibility Criteria

Studies evaluating the use of minimally invasive or scarless techniques for aesthetic reconstruction were deemed eligible for inclusion. Minimally invasive or scarless techniques were defined by having no visible anterior breast scars except periareolar. Studies were included if they met the following additional criteria: human subjects, performed for aesthetic or reconstruction purposes, and reported at least one measurable outcome such as BREAST-Q assessment, patient-reported satisfaction, visual cosmetic assessment, etc. 

Excluded studies were traditional mastopexy techniques with visual anterior breast scars, animal or cadaveric studies, and those without a measured tool to assess outcomes defined as lacking necessary data for statistical analysis. Additionally, case reports were not considered for inclusion. 

Study Selection

Two reviewers (J.G. and N.B.) independently screened titles and abstracts for eligibility. Full-text articles deemed relevant were then reviewed independently by both reviewers in detail against the inclusion and exclusion criteria to assess final eligibility. Discrepancies were resolved by discussion with a third and fourth reviewer (E.R.S. and C.S.). The screening process was conducted utilizing Rayyan AI-Powered Systematic Review Management Platform (Cambridge, MA, USA). 

Data Collection Process

Two reviewers (J.G. and N.B.) independently extracted data that met the final inclusion criteria using a standardized form, recording article title, author and year of publication, patients in assessed cohort, number of breast(s) operated on, mean age and BMI, surgical procedure and technique, operative time (minutes), reported outcomes (i.e. BREAST-Q, patient reported satisfaction, cosmetic score, Harvard Scale), associated complications (i.e., hematoma, capsular contracture, implant malposition, dehiscence), revisions required, and recovery time. When multiple outcome measures were reported, priority was given to validated assessment tools. 

Risk of Bias Assessment

A thorough examination of the risk of bias and certainty of evidence was assessed independently by two reviewers (J.G. and N.B.) through a methodical review of each study’s design, patient selection, outcome measurement, missing data, and reporting criteria. Study validity and robustness of evidence were confirmed independently by two additional reviewers (E.R.S. and C.S.). Assessing the literature with structured criteria allowed for a uniform comparison across all evaluated studies. Due to the majority of available literature on minimally invasive and scarless breast surgery having been conducted almost exclusively in non-randomized, single-armed studies, a degree of confounding bias and randomized patient selection practices are unavoidable. In the available literature, the absence of a control group is inherent and treatment placement is therefore limited. Additionally, this finding is inevitable in aesthetic surgery where randomization of procedure type is impractical. Our data synthesis was therefore focused on qualitative outcomes and included quantitative variables only when parallels between could be drawn with minimal heterogeneity. Presenting the synthesized data in this manner allows for the outcomes to be demonstrated as valuable clinical experience while maintaining transparency regarding the methodical limitations native to the assessment of these non-randomized studies.

Statistical Analysis

This review provides a quantitative synthesis of outcomes for scarless techniques for aesthetic breast procedures, standardizing comparisons across related studies. Additional subjective aesthetic outcomes were synthesized descriptively. Variables including nipple lift or areolar diameter reduction were reported as means when provided, and categorical variables such as complications and revision rates were expressed as percentages. When individual studies reported comparable outcome measures, pooled means were calculated according to the sample size of each study. Recognizing the heterogeneity of study designs, surgical techniques, and outcome measures, a meta-analysis was not performed.

Results

Study Selection

**Figure 1:**
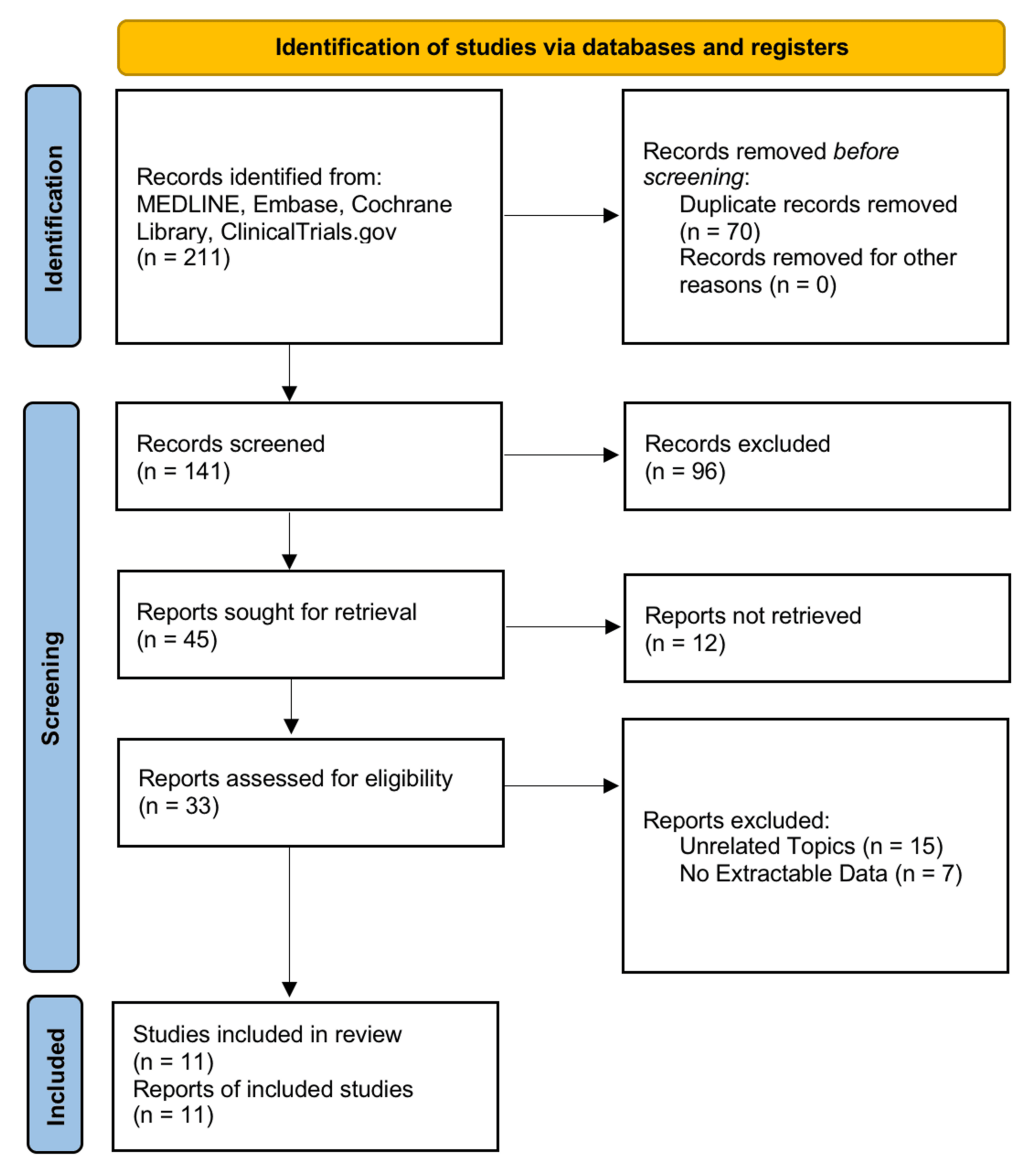
Preferred Reporting Items for Systematic Reviews and Meta-Analyses (PRISMA) flow diagram outlining the systematic review process, including identification, screening, eligibility, and inclusion of studies.

Database searching identified 211 unique records. After title and abstract screening, 33 full-text articles were assessed for eligibility. Of these, 11 studies met the inclusion criteria. Twenty-two articles were excluded: due to the investigation of unrelated topics (e.g., oncologic or reconstruction-focused outcomes) [20-33], and for reporting no extractable measured outcomes (e.g., no mention of BREAST-Q, cosmetic scoring, or patient-reported satisfaction) [34-40]. The study selection process is illustrated in Figure 1.

Study Characteristics

**Figure 2:**
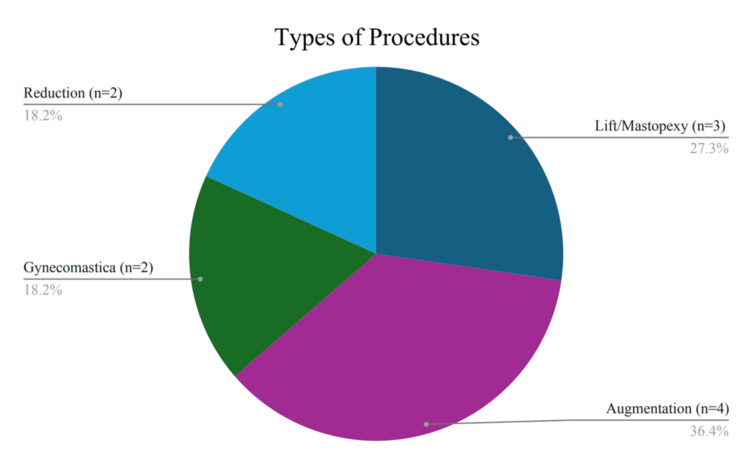
Categorization of Scarless Procedures Distribution of included scarless breast and chest procedures, showing the relative proportions of augmentation, mastopexy, reduction, and gynecomastia techniques [12,13,41-49].

**Figure 3:**
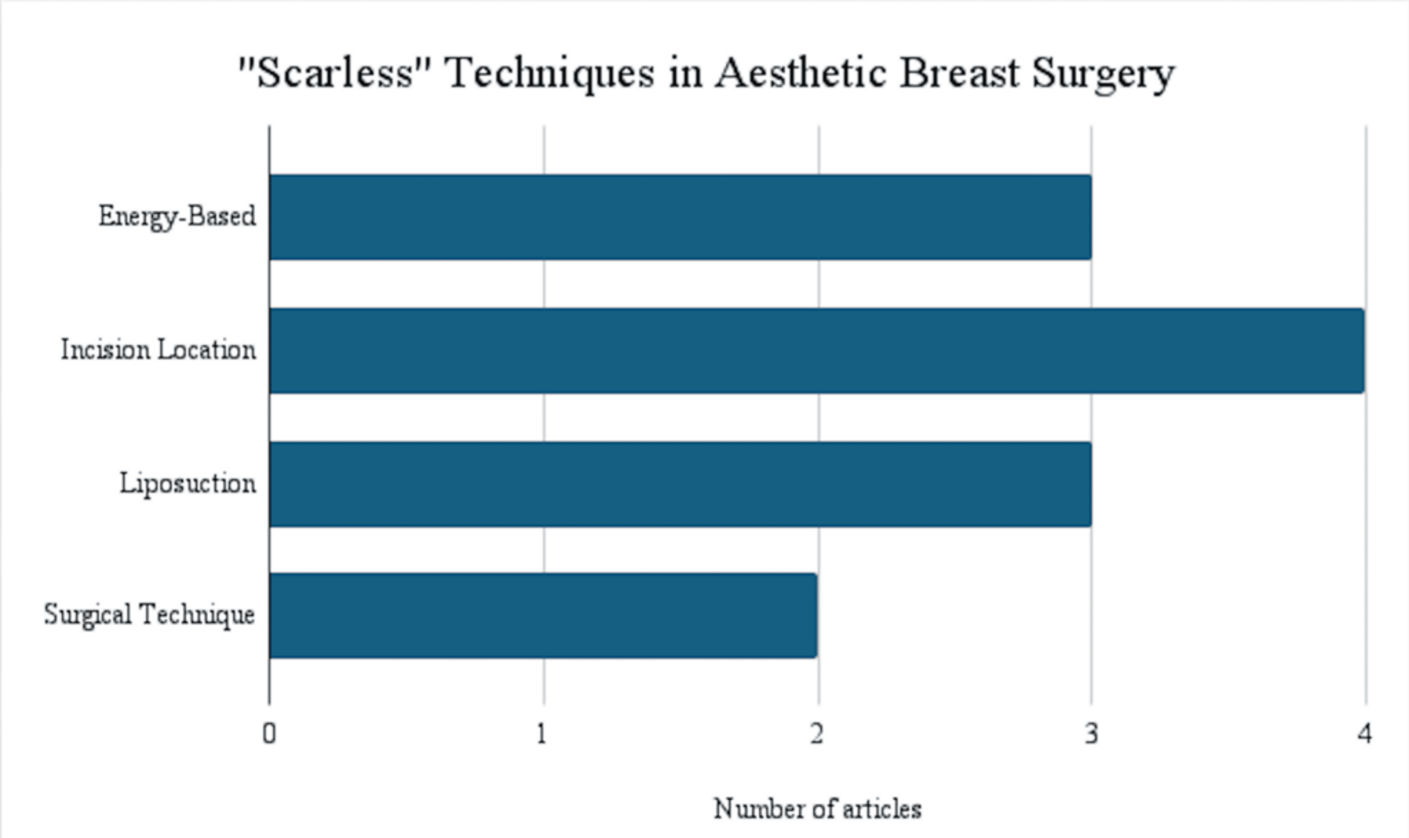
Categorization of Scarless Techniques Categorization of scarless surgical methods across included studies, grouped by energy-based, incision location, liposuction-based, and surgical innovations [12,13,41-49].

The included studies explored scarless breast surgery as alternatives to standard procedures including breast augmentation (n=4), breast lift/mastopexy (n=3), breast reduction (n=2), and gynecomastia (n=2) as illustrated in Figure 2. Given this substantial heterogeneity in surgical goals, techniques, and outcome measures across aesthetic breast procedures, results were stratified by procedure type. This approach allows for more clinically meaningful comparisons of scarless techniques within each procedural category, as differences in anatomy, operative objectives, and outcome metrics limit direct comparison across procedure types. While various techniques and methods to achieve “scarless” results, the most common included alternative incision location (n=4), energy-based procedure (n=3), use of liposuction (n=3), and unique surgical technique (n=2) as illustrated in Figure 3 [12,13,41-49]. 

Breast Augmentation

Four studies in this review evaluated techniques aimed at minimizing or concealing the classic scars of breast augmentation, including scarless fat grafting with dual-anchor cog threads [41], liposuction-augmentation mammaplasty [42], transumbilical augmentation with silicone implants [43], and endoscopic-assisted transaxillary augmentation [44] as seen in Table 1. Across these studies, patient enrollment ranged from 22 to 125, with mean ages between 26.3 and 37 years when reported. Operative times varied considerably, from 46 minutes in the liposuction-augmentation cohort to 90 minutes in the transumbilical approach. Patient satisfaction was highest in the scarless fat grafting and transumbilical groups, though assessment tools differed. Visconti et al. [41] employed the BREAST-Q and reported overwhelmingly positive ratings (nine excellent, 20 very good, five good). Lee et al. [43] used a patient-reported scale but presented incomplete data, summarizing results broadly as “high satisfaction.” Harris et al. did not use a validated scale, while the transaxillary study emphasized safety outcomes rather than aesthetic metrics.

**Table 1:** Summary of scarless and scar-minimizing breast augmentation techniques

Study / Author	Patients (n)	Mean Age (yrs)	BMI	Technique Category	Operative Time (min)	Aesthetic Outcomes	Results	Complications	Revision
Visconti & Salgarello [41]	22	26.3	26.5	Scarless / Fat grafting	n/a	BREAST-Q	9 Excellent, 20 Very good, 5 Good	None	none
Harris et al. [42]	125	37	24	Liposuction + Implant	46	No scale	Satisfaction not formally measured	Asymmetry (2), Capsular contracture (2), Malposition (1)	2.50%
Lee et al. [43]	42	31.6	-	Incision – Umbilical	90	Patient-reported (0–4 scale, incomplete data)	Reported “high satisfaction”	Bulging (5), Hypertrophic scar (1), Contracture conversion (3), Scar widening (4)	4 patients
Kolker et al. [44]	197	-	-	Incision – Axillary	-	Not specified	Generally safe; emphasis on technique refinements	Conversion to open: 0.5% (bleeding), 1.5% (positioning). Revisions: 3.5% (malposition)	Yes

Complications reflected the trade-off between incision type and morbidity. Scarless fat grafting was notable for reporting no complications. Liposuction-augmentation carried a modest but clinically relevant risk of capsular contracture (n=2), asymmetry (n=2), and implant malposition (n=1). The transumbilical approach demonstrated the highest complication burden, including periumbilical bulging, hypertrophic scarring, capsular contracture necessitating conversion, and scar widening. The transaxillary series reported low complication rates, though 0.5% required conversion to open surgery for bleeding, 1.5% for implant positioning, and 3.5% underwent revision for malposition. Revision rates were lowest in scarless approaches and more common in incision-based methods. Overall, alternative augmentation techniques achieved high satisfaction and acceptable safety, but incision-based approaches were associated with scar-related complications, displaying the balance between cosmetic benefit and surgical risk.

Breast Lift/Mastopexy

Three studies in this review examined alternative mastopexy methods aimed at reducing or eliminating traditional scars as seen in Table 2. These included helium-based plasma radiofrequency, radiofrequency-assisted lipolysis, and the intra-areolar “Compass Rose” suture technique [45]. Patient enrollment ranged from 10 to 77, with mean ages spanning 29.7 to 39 years. Across techniques, both objective anthropometric measurements and patient-reported outcomes demonstrated favorable results. For instance, Sterodimas et al. documented measurable decreases in nipple position distances with significant improvements on the BREAST-Q and Global Aesthetic Improvement Scales, while Unger reported statistically significant improvements in nipple-to-fold and nipple-to-nipple distances sustained up to 12 months [12,13,46].

**Table 2:** Summary of scarless mastopexy techniques GAIS: Global Aesthetic Improvement Scale, SN-N: sternal notch-to-nipple distance, IPR: independent photographic review, NAC: nipple-areola complex, IMF: inframammary fold

Study / Author	Patients (n)	Mean Age (yrs)	BMI	Technique Category	Measurement of Aesthetic Outcomes	Results	Complications
Sterodimas et al. [12]	15	38	21.5	Energy-based	I-GAIS, P-GAIS, BREAST-Q	Measured nipple lift (-1.2 cm SN-N), 11/15 IPR success, +1.29 BREAST-Q improvement	None
Unger [13]	10	39	-	Energy-based	Patient satisfaction, breast measurements	NAC reposition maintained at 12 months; ↓ nipple-IMF distance (p=0.03–0.05), ↓ nipple-nipple distance (p=0.01)	Not reported
Ionescu et al. [45]	77	29.7	19.4	Suture/Incision	Oral questioning, Northwood Index (NI)	Areolar width ↓11.3 mm (20%), NI improved (0.55 → 0.36); 2 dissatisfied (projection)	Hematoma (2), hypersensitivity (3), knot migration (1), recurrence (1)

Satisfaction rates were generally high, though assessment tools varied. The plasma- and radiofrequency (RF)-assisted techniques reported strong improvements without complications, while the intra-areolar pexy showed favorable reshaping of areolar herniation and tuberous deformity but carried modest risks. Complications in the Compass Rose cohort included hematoma, nipple hypersensitivity, suture knot migration, and recurrence in isolated cases, whereas energy-based studies reported none. Revision rates were low overall, with the best safety and satisfaction observed in energy-based approaches. These findings highlight energy-based mastopexy as a promising scar-minimizing alternative, while suture-based methods may remain useful for tuberous breast correction despite slightly higher complication rates.

Breast Reduction

Two studies evaluated breast reduction techniques aimed at minimizing visible scars as seen in Table 3. Abboud et al. described a no-scar method combining power-assisted liposuction, loops, and lipofilling [47], while Echo et al. assessed a no-vertical-scar inferior pedicle reduction using a dermal suspension sling [48]. Sample sizes varied, with Abboud et al. reporting 94 patients and Echo et al. reporting 38, with mean ages of 37 and 38 years, respectively. The liposuction-based approach yielded strikingly high satisfaction, with 97% of patients pleased with breast shape, 97% willing to repeat the procedure, and 95% satisfied with postoperative pain. In contrast, the dermal sling study did not use a formal scale, but follow-up impressions suggested favorable outcomes.

**Table 3:** Summary of scarless breast reduction techniques

Study / Author	Patients (n)	Mean Age (yrs)	BMI	Technique Category	Aesthetic Outcomes	Results	Complications	Recovery
Abboud et al. [47]	94	37	29	Liposuction / Energy-based	Patient survey	97% satisfied with shape; 97% would repeat; 95% satisfied with pain	Cellulitis (1)	n/a
Echo et al. [48]	38	38	34.5	Surgical (Dermal sling)	No scale	Favorable results noted on follow-up	Wound dehiscence (1), Infection (2), Fat necrosis (1)	3 days

Complications were infrequent across both methods but more common in the dermal sling technique. Abboud et al. reported only one case of cellulitis, whereas Echo et al. described wound dehiscence, infections, and fat necrosis in a small subset of patients. Recovery times were not detailed in Abboud et al.’s study but averaged three days for the dermal sling technique. Overall, liposuction-based reduction provided the highest patient satisfaction with minimal morbidity, while the dermal sling method offered another scar-minimizing approach, though with a slightly greater risk of wound complications.

Gynecomastia

Two studies investigated techniques for reducing scars in gynecomastia surgery as seen in Table 4. Luo et al. presented a large single-institutional series of 1,082 patients treated with a scar-hidden endoscopic technique [49], while Sarkar et al. evaluated 12 patients with high-grade disease managed using combined circumareolar excision and liposuction [50]. The endoscopic cohort had a mean age of 37.6 years, while the smaller series reported a mean age of 25.8 years. Luo et al. reported a remarkably short mean operative time of 17.7 minutes, with universal patient satisfaction. Sarkar et al. also documented strong satisfaction, with five patients reporting excellent results and seven reporting good results.

**Table 4:** Summary of scarless gynecomastia techniques

Study / Author	Patients (n)	Mean Age (yrs)	Technique Category	Operative Time (min)	Measurement of Aesthetic Outcomes	Results	Complications
Luo et al. [49]	1082	37.6	Endoscopic / Scar-hidden	17.7	Patient satisfaction	100% satisfaction	Epidermal necrosis (7), Ecchymosis (4)
Sarkar et al. [50]	12	25.8	Liposuction + Excision	n/a	Patient satisfaction	5 Excellent, 7 Good	Seroma (3), Hematoma (1)

Complications were uncommon but varied between approaches. In Luo et al.’s large cohort, epidermal necrosis occurred in seven patients and ecchymosis in four, representing a low incidence relative to the study size. Sarkar et al.’s smaller series reported three cases of seroma and one case of hematoma, with an average length of stay of 2.4 days. Neither study specified revision rates. Taken together, endoscopic scar-hidden gynecomastia surgery demonstrates exceptional safety, efficiency, and satisfaction in large-scale application, while combined circumareolar excision and liposuction provides a feasible alternative for high-grade disease with slightly higher risks of fluid collection.

Discussion

Scarless and scar-minimizing methods in breast and chest surgery are increasingly recognized as viable alternatives to conventional techniques, particularly for patients with strong aesthetic concerns regarding visible scarring. While traditional augmentation, mastopexy, and reduction procedures achieve durable shape and volume improvements, they often leave conspicuous scars that can affect psychosocial well-being. In this review, scarless and scar-hidden approaches demonstrated comparable, and in some cases superior, outcomes in terms of patient satisfaction, though complication patterns varied depending on the technique.

Efficacy of Scarless Versus Conventional Approaches

Conventional implant augmentation remains durable but is constrained by incision-related risks, most notably capsular contracture (CC). A meta-analysis found periareolar incisions carry a higher CC risk than inframammary (OR~1.9), with no clear difference versus transaxillary access, reinforcing inframammary fold (IMF) as the benchmark scar placement for reducing CC [51]. Complementary reviews echo lower CC risk with IMF compared with periareolar approaches [52]. In contrast, the scarless fat-grafting + dual-anchor cog thread method reported uniformly high BREAST-Q satisfaction with no thread-related complications, indicating a favorable risk-benefit profile when modest volume augmentation and footprint shaping suffice. Scar-relocating strategies reduce visible breast scars but add access-specific trade-offs: transumbilical subpectoral augmentation achieved high satisfaction yet showed periumbilical bulging, hypertrophic scarring, and occasional conversion for capsular contracture.

In traditional mastopexy, pedicle choice influences outcomes. A 5123-breast meta-analysis reported superomedial pedicles yield shorter operative time and higher BREAST-Q satisfaction, with lower infection but higher seroma and reduced NAC sensation versus inferior pedicles, underscoring predictable trade-offs in conventional lifts [53]. Compared head-to-head with our “scarless” cohort, energy-based lifts (helium plasma; radiofrequency-assisted lipolysis) improved nipple position and contour with no or minimal adverse events in prospective series, while the intra-areolar “Compass Rose” pexy effectively corrected areolar herniation/tuberous features but at the cost of occasional hematoma, hypersensitivity, and recurrence. 

Evidence for conventional reduction shows technique-dependent differences rather than a single best approach. A recent meta-analysis comparing superomedial vs. inferior pedicles found higher BREAST-Q satisfaction and shorter operative length with superomedial pedicles but increased seroma and reduced NAC sensation - again highlighting a balance between efficiency and sensory risk [54]. In comparison, liposuction-dominant reduction (power-assisted liposuction + loops/lipofilling) reported very high satisfaction and ~1% total complications (single cellulitis) across a sizable series, suggesting that for moderate hypertrophy, minimally invasive reduction can approach or surpass conventional patient-reported outcomes while lowering wound-morbidity exposure. 

For traditional management, a comprehensive review synthesizing practice patterns supports excision ± liposuction tailored to grade, with hematoma/seroma the most frequent complications; a literature review of complication incidence suggests that combined excision + aspiration may reduce complications versus excision alone [55]. Against this benchmark, the endoscopic scar-hidden approach in a >1,000-patient series achieved near-universal satisfaction, very short operative times (~18 min), and low complication rates, indicating scalability and safety when tissue quality and grade allow; circumareolar skin excision and liposuction remains useful for high-grade disease but with higher fluid-related events.

“*Scarless” Techniques*

Across all categories, four broad strategies emerged in the pursuit of scarless surgery: energy-based techniques (helium plasma, RF-assisted lipolysis), liposuction-based approaches (with or without fat grafting), innovative surgical techniques (intra-areolar pexy, dermal suspension slings), and incision relocation (transumbilical, transaxillary). Each has distinct strengths and limitations: energy-based methods offered reproducible results with minimal morbidity; liposuction provided quick, minimally invasive reductions but did not eliminate risks of contour irregularity or capsule-related issues; surgical techniques reduced scar length but retained wound-related risks; and incision relocation effectively concealed scars while introducing access-specific complications.

Emerging Front-Runners in Practice 

Among these modalities, certain front-runners emerged. Endoscopic scar-hidden gynecomastia surgery and no-scar liposuction-based breast reduction showed the strongest combination of reproducibility, scalability, and safety, supported by large cohorts and high satisfaction. Energy-based mastopexy also holds promise for broader adoption as technology advances. In augmentation, scarless fat grafting with cog threads demonstrated compelling early results but requires further validation in larger, controlled studies.

Patient Selection and Indications

The success of these methods depends heavily on patient selection. Energy-based mastopexy is best suited for patients with mild to moderate ptosis and good skin quality, while liposuction-based reduction is appropriate for moderate hypertrophy. Scarless fat grafting is ideal for patients desiring modest volume enhancement without large implants. Endoscopic gynecomastia techniques work best in low- to moderate-grade disease, whereas excisional methods remain necessary for severe or fibrotic cases. These observations highlight that scarless techniques are not universally superior but rather expand the surgical armamentarium, allowing for more individualized approaches.

Ultimately, the adoption of scar-minimizing approaches requires careful balancing of aesthetic priorities against complication risk and long-term durability. While scarless methods consistently demonstrated excellent patient satisfaction, incision-based modifications often shifted rather than eliminated complications. Standardized reporting using validated instruments such as the BREAST-Q is needed to facilitate objective comparisons across studies. Future research should focus on randomized controlled trials, long-term durability data, and integration of regenerative strategies to further optimize outcomes. Scarless techniques, when applied to the right patient populations, are poised to complement, and maybe even replace, conventional approaches, offering individualized solutions that prioritize both safety and aesthetics.

Limitations and Future Direction

This review is limited by the heterogeneity of pooled aesthetic breast surgery techniques throughout studies and further constrained through outcome measures. Standardized instruments such as the BREAST-Q were used infrequently, and many reports depended on subjective evaluations such as global aesthetic scales and photographic reviews. These findings serve to emphasize that in order to more thoroughly determine the effectiveness and safety of scarless breast surgery, prospective trials with consistent reporting are required.

## Conclusions

Scarless breast surgery techniques regularly yield satisfactory patient outcomes and significant improvements in nipple position and breast contour, with complication rates similar to those of traditional approaches. While traditional approaches to augmentation, mastopexy, and reduction reliably achieve both volume and shape improvements, they frequently leave behind incisions that not only are aesthetically compromised, but also may impact patient well-being. These newer, less invasive methods demonstrate the most promise when patients value shorter recovery timeframes and minimal scarring. Emerging front runners, such as liposuction-based reduction, endoscopic scar-hidden gynecomastia surgery, and energy-based mastopexy consistently demonstrate their utility against larger patient cohorts. Clinically, surgeons can achieve aesthetic goals with scarless operations while ensuring consistent results. Standardized reporting, longer-term follow-up, and comparative research trials to determine the significance of minimally invasive procedures in influencing cosmetic breast practice will be beneficial moving forward.
